# Feasibility and Safety of Primary Ureteroscopy with Single-Use Flexible Ureteroscope HU30M (6.3 Fr, HugeMed): An Initial Experience

**DOI:** 10.3390/diagnostics15192522

**Published:** 2025-10-05

**Authors:** Benedikt Ebner, Iulia Blajan, Johannes Raphael Westphal, Iason Papadopoulos, Troya Ivanova, Deniz Karatas, Moritz Happe, Yannic Volz, Christian G. Stief, Maria Apfelbeck, Michael Chaloupka

**Affiliations:** Department of Urology, University Hospital of the LMU Munich, 81377 Munich, Germany

**Keywords:** primary ureteroscopy, flexible ureteroscopy, disposable ureteroscope, 6.3 Fr

## Abstract

**Background:** The miniaturization of ureterorenoscopes increasingly enables atraumatic primary ureteroscopy, without ureteral dilation or presenting. This study aims to evaluate the feasibility and safety of primary ureteroscopy using the HU30M (6.3 Fr, HugeMed, Shenzhen HugeMed Medical Technical Development Co., Ltd., Shenzhen, China), the smallest currently available ureteroscope. **Methods:** We analyzed consecutive patients in whom primary ureteroscopy using the HU30M was performed or attempted, using prospectively collected in-hospital and 30-day follow-up data for retrospective evaluation. The primary outcome was the success rate of primary ostial intubation. Secondary outcomes included the stone-free rate (SFR) in patients with urolithiasis, incidence of in-hospital complications (Clavien–Dindo classification) and 30-day emergency readmission. Additionally, we conducted a propensity score-matched comparative analysis of the HU30M versus a contemporary 7.5 Fr digital single-use ureteroscope (PUSEN PU3033AH, Zhuhai Pusen Medical Technology Co., Ltd., Jinhua, China). **Results:** Between January and April 2025, primary ureteroscopy using the HU30M was performed or attempted in 34 patients, including four bilateral procedures. Primary ureteroscopy was defined as ureteroscopic access without prior stenting or dilation. Indications were diagnostic evaluation in 15 patients (44%), uretreroscopic stone treatment in 10 patients (29%) and endoscopic combined intrarenal surgery (ECIRS) in 9 patients (27%). Successful primary ostial intubation was achieved in 36 of 38 renal units (95%). Among urolithiasis cases, SFR was 17/19 (90%) in-hospital complications were limited to postoperative fever in two patients (6%) and no procedure-related 30-day emergency readmission occurred. In matched analyses, HU30M demonstrated significantly shorter operative times compared with the 7.5 Fr ureteroscope, while postoperative hemoglobin drop, inflammatory parameters and renal function were comparable. **Conclusions:** Primary ureteroscopy with HU30M is feasible and safe across diverse indications, achieving high success of atraumatic ostial access. Comparative analyses suggest procedural efficiency advantages and overall safety comparable to the current digital single-use ureteroscope standard.

## 1. Introduction

Technical advances in instrument miniaturization improved the quality of ureterorenoscopy [1]. Multiple studies demonstrate that, in general, smaller-diameter instruments are associated with fewer perioperative and long-term complications compared to larger devices [2,3]. Nevertheless, many patients undergo preoperative ureteral stent placement for several days before ureterorenoscopy. Studies show that this prestenting reduces complication rates compared to ureterorenoscopy performed without prior stenting [4,5]. The advent of ultra–thin ureterorenoscopes shows the potential of safe primary ureterorenoscopy without prestenting, therefore shortening the time to definitive intervention or diagnosis, reducing the number of procedures, and lowering overall healthcare costs [6,7]. The single-use HU30M (Shenzhen HugeMed Medical Technical Development Co., Ltd., Shenzhen, China), at 6.3 Fr, is currently the thinnest ureterorenoscope available. Compared with standard reusable or single-use ureteroscopes of 7.5–9 Fr, the HU30M combines a super-slim outer diameter of 6.3 Fr with a standard 1.2 mm working channel, thereby maintaining compatibility with commonly used endourological instruments. The bullet-shaped distal end and the flexible insertion tube are specifically designed to facilitate atraumatic intubation of the ureteral orifice and to reduce the need for pre-stenting or active dilation.

These design features show a potential to translate into several clinical advantages over conventional devices. By lowering the requirement for passive or active ureteral dilation, the HU30M may reduce the risk of access-related complications such as ureteral trauma or postoperative discomfort. The ability to perform safe primary ureteroscopy without prestenting could shorten the time to definitive treatment, reduce the number of required interventions, and ultimately lower healthcare costs.

In the present study, we evaluated the feasibility and safety of primary ureteroscopy using the HU30M across various clinical indications, with particular focus on primary ostial intubation success rates, stone-free rates, and perioperative complications. Furthermore, we present a propensity score-matched comparison of stone treatment by ureterorenoscopy (6.3 Fr HU30M vs. 7.5 Fr) and endoscopic combined intrarenal surgery (ECIRS HU30M vs. ECIRS 7.5 Fr).

## 2. Materials and Methods

### 2.1. Study Design and Patient Cohort

We performed an analysis of 34 consecutive patients in whom primary ureteroscopy using the HU30M was performed or attempted between January and April 2025 at the Department of Urology, LMU University Hospital Munich, Germany. The HU30M is a single-use digital flexible ureteroscope with an outer diameter of 6.3 Fr and a working length of 650 mm. The instrument provides 285° up-and-down deflection, a 120° field of view, and contains a 1.2 mm working channel. The scope features a slim insertion tube, increased maneuverability, and a bullet-tipped distal end to facilitate smooth and atraumatic insertion. The HU30M is a single-use digital instrument and not supposed to be resterilized. Therefore, each device was used only once. In bilateral cases, the same scope was employed for both renal units during the same operation, but no device was reused across different patients or procedures. All patients with an indication for primary ureteroscopy who provided informed consent underwent the procedure and were included in our analysis. Primary ureteroscopy was defined as procedures performed without prior stenting or intraoperative ureteral dilatation. For ureterorenoscopic (URS) access of the upper urinary tract, we used a four-step, escalating strategy. After freehand cystourethroscopy, we first attempted primary ureteral intubation without probing the ureteral orifice with the URS (no-touch technique). If this was unsuccessful owing to the orifice angle or the intramural ureter, we cannulated the orifice and ureter with a guidewire via the URS and then re-introduced the scope, attempting parallel advancement alongside the in situ wire. If intubation remained unsuccessful, a guidewire was advanced through the working channel into the orifice, and the URS was railroaded over the wire to achieve atraumatic access. As a final step, we attempted combined parallel-and over-the-wire intubation (“railway” technique). If access was still not feasible, the primary URS was aborted and a ureteral stent was placed for pre-stenting. No additional inclusion criteria were applied. For laser lithotripsy, a pulsed thulium-YAG laser (Thulio, Dornier MedTech GmbH Wessling, Germany) was used. No ureteral access sheaths were used. For patients who underwent endoscopic combined intrarenal surgery (ECIRS), a minimally invasive percutaneous nephrolithotomy (MIP) system from Karl Storz GmbH & Co. KG, Lecce, Italy, was utilized. Percutaneous access for ECIRS was performed using triple imaging guidance consisting of endoscopy, ultrasound and fluoroscopy. Following primary ureteroscopy and an initial endoscopic inspection of the collecting system, a urography was performed by instilling a mixture of contrast medium and indigocarmine blue dye through the endoscope’s working channel. This was followed by ultrasound-guided puncture of the collecting system, with endoscopic confirmation of correct needle placement prior to guide wire insertion. After inserting the guide wire, sequential tract dilation was performed under fluoroscopic and endoscopic guidance. No ureteral catheters were used. According to current guidelines, patients with sterile urine receive a single intraoperative dose of a second-generation cephalosporin. Patients with a positive urine culture underwent targeted perioperative antibiotic therapy. No patient in the study cohort was under specific pharmacological metaphylaxis—such as HCT, calcium, magnesium, alkalizing citrate preparations, or xanthine oxidase inhibitors—prior to enrollment. A ureteral stent was placed routinely after primary ureteroscopy, in some cases on a string, at the discretion of the treating surgeon. Patients remained hospitalized for a minimum of one night for observation after primary ureteroscopy. Stone-free status was evaluated by intraoperative endoscopic and fluoroscopic assessment and postoperatively by renal ultrasound performed within 3 days after surgery. On the first postoperative day, laboratory tests were conducted for all patients. Patient data and clinical outcomes were collected prospectively during hospitalization and through a telephone follow-up after 30 days. For the present study, the prospectively collected data were analyzed retrospectively.

In order to provide a contemporary control group, the original study cohort was expanded by an additional 23 patients undergoing ureteroscopic stone treatment with the standard of care of our department, PU3033AH PUSEN (Zhuhai Pusen Medical Technology Co., Ltd., Zhuhai, China) ureteroscope, between October and December 2024. The PUSEN PU3033AH is a single-use, flexible, digital ureteroscope with a 7.5 Fr outer diameter and a 3.6 Fr working channel, designed for a 650 mm working length and featuring a 270° dual-bending tip. Of these 23 patients, 14 underwent ureteroscopic stone treatment alone (URS PUSEN) and 9 underwent endoscopic combined intrarenal surgery (ECIRS PUSEN). All patients included in this analysis had undergone prestenting before surgery, in contrast to the HU30M cohort, which was treated as primary ureteroscopy or ECIRS without prestenting. A 12/14 Fr ureteral access sheath was used in 7 of the 14 URS PUSEN cases. In 20 of the 23 cases, a postoperative ureteral stent was placed. All patients provided prior informed consent. Perioperative management, including handling of preoperative urine culture results, antibiotic therapy, postoperative laboratory testing, and ultrasound follow-up, was conducted identically in both groups.

The study adhered to the ethical principles of the Declaration of Helsinki and was approved by the University Ethics Committee (#22-0949, date of approval: 13 January 2023).

### 2.2. Outcomes

The primary outcome was the success rate of primary ostial intubation (i.e., successful access to the upper urinary tract without prior prestenting or dilation). Secondary outcomes were stone-free rate of patients with urolithiasis (defined as residual fragments <1 mm, assessed endoscopically at the end of the procedure), the incidence of in-hospital complications (Clavien–Dindo classification) and procedure-related emergency readmission rate within 30 days of discharge.

### 2.3. Statistical Analysis

Continuous variables are presented as medians with interquartile ranges (IQR), and categorical variables are presented as proportions. Propensity score matching based on maximal stone diameter was applied separately for URS (HU30M vs. PUSEN) and ECIRS (HU30M vs. PUSEN) cohorts using a 1:1 nearest-neighbor algorithm. Continuous variables are reported as median (interquartile range) and compared with the Wilcoxon rank-sum test. A two-sided *p* value < 0.05 was considered statistically significant. The statistical analyses were performed using DATAtab Team (DATAtab e.U., Graz, Austria) and R (version 4.5.0; R Foundation for Statistical Computing, Vienna, Austria).

## 3. Results

### 3.1. Baseline Characteristics and Indications for Primary Ureteroscopy

Patient characteristics are displayed in Table 1. Between January and April 2025, primary ureteroscopy using the HU30M ureteroscope was performed or attempted in 34 patients at our department. The median age of the study population was 62 years (IQR 46–71), with an equal gender distribution (50% male, 50% female). The median BMI was 27 kg/m^2^ (IQR 22–30), and 41% of patients had an ASA class >2. There were three indications for primary ureteroscopy, which were distributed as follows: (1) diagnostic evaluation in 15 patients (44%), (2) stone treatment using ureteroscopy alone in 10 patients (29%), and (3) endoscopic combined intrarenal surgery (ECIRS) in 9 patients (27%).

### 3.2. Success Rate of Primary Ostial Intubation

Of the 34 patients included, four underwent bilateral primary ureteroscopy, giving a total of 38 renal units in which primary ureteroscopy was either performed or attempted. Table 2 shows the approach of ostial intubation. Successful primary ostial intubation was achieved in 36 of 38 renal units (95%). Four techniques were employed: (1) no-touch technique without any guidewire in 11 cases (29%), (2) one guidewire parallel to the ureteroscope in 5 cases (13%), (3) one guidewire through the ureteroscope in 9 cases (24%), and (4) two guidewires (railway technique) in 11 cases (29%). Only 2 cases (5.3%) were recorded as intubation failure. Both patients were young women (16 and 25 years old). The 16-year-old patient (BMI 21, ASA II) underwent surgery for two renal stones measuring 5 and 6 mm in maximum diameter. The previously described four-step intubation technique was unsuccessful, and the procedure was aborted after 31 min with placement of a ureteral stent for pre-stenting. In the second case, a 25-year-old patient (BMI 19, ASA II) underwent diagnostic URS for unexplained hydronephrosis. Atraumatic intubation again proved unsuccessful, and the procedure was concluded after 40 min with pre-stenting.

### 3.3. Stone-Free Rates and Stone Characteristics

In 10 patients with low stone burden, we performed primary ureteroscopy using the HU30M. Stone-free status was achieved in 9 of 10 patients (90%) following the procedure. The median number of stones was 2 (IQR 1–2) per patient. The median diameter of the largest stone was 6 mm (IQR 4–6) in the coronal plane, 5 mm (IQR 4–5) in the sagittal plane, and 4 mm (IQR 4–5) in the transversal plane. The median radiodensity of the largest stone was 718 Hounsfield Units (HU) (IQR 337–897). The median operative time was 31 min (IQR 29–43). Stone analysis was performed in 15 of 19 patients (78.9%). Pure calcium oxalate (monohydrate and/or dihydrate) comprised 60% of analyzed stones, while 40% consisted of mixed compositions containing calcium oxalate combined with carbonate apatite or uric acid.

Nine patients underwent primary ureteroscopy using the HU30M as part of an endoscopic combined intrarenal surgery (ECIRS). The stone-free status in the ECIRS group was 89% (8/9 patients). Patients in this group presented with a median of 2 stones per patient (IQR 1–4). The median diameter of the largest stone was 15 mm (IQR 13–16) in the coronal plane, 14 mm (IQR 11–18) in the sagittal plane, and 14 mm (IQR 11–15) in the transversal plane. The median radiodensity was 830 HU (IQR 714–967). The median Guy’s stone score was 3 (IQR 2–3). The median operative time was 57 min (IQR 42–63).

### 3.4. Incidence of In-Hospital Complications and 30-Day Readmission Rate

The incidence of in-hospital complications was 6% (2/32 patients). Fever was the only Clavien–Dindo complication observed in this study, with no other patients developing complications during their hospital stay (Table 1). Specifically, two patients who underwent ECIRS developed postoperative fever classified as Clavien–Dindo II. Both had positive preoperative urine cultures—one with Proteus mirabilis and one with E. coli—and received appropriate antibiotic therapy before surgery. Both patients were treated for a staghorn stone spanning multiple calyces. In both cases, the fever resolved by the second postoperative day. Neither patient exhibited additional signs of systemic infection, such as tachycardia, tachypnea, hemodynamic instability, or other features of urosepsis. No major complications (Clavien–Dindo ≥ IIIb) were recorded in either group.

Furthermore, no procedure-related 30-day emergency readmissions occurred.

### 3.5. Additional Perioperative Finding

The median operation time was 43 min (IQR 29–57), with the longest median duration observed in the ECIRS group (57 min (IQR 42–63)). Postoperative ureteral stenting was performed in 97% of all cases. In patients who did not require further treatment, the ureteral stent was removed after a median of 7 days (IQR 2–14). In one patient (ECIRS), the insertion of a ureteral stent at the end of the surgery was not possible due to increased resistance encountered during stent placement, presumably resulting from post-operative ureteral edema. The patient received a postoperative nephrostomy, which was removed after 4 days without further complications.

Postoperative laboratory parameters demonstrated minimal median changes from preoperative baseline values (Table 3). The median hemoglobin level decreased slightly by −0.8 g/dL (IQR −1.3, −0.1), while median leukocyte count increased by 2.5 G/L (IQR 0.4–4.7). C-reactive protein showed a minor median elevation of 0.4 mg/dL (IQR 0.1–0.6). Renal function parameters remained stable, with no significant changes in median creatinine levels and estimated glomerular filtration rate (eGFR).

### 3.6. Comparative Analyses with Standard of Care 7.5 Fr Digital Single-Use Ureteroscope

To address potential selection bias and provide a contemporary control group, the original patient cohort was extended by 23 additional patients treated with the standard of care at our department, 7.5 Fr PUSEN PU3033AH ureteroscope (Zhuhai Pusen Medical Technology Co., Ltd., Zhuhai, China). After propensity score matching for maximal stone diameter, 10 patients were available in each Ureteroscopy group and 9 patients in each ECIRS group. Patient characteristics of the updated cohort are displayed in Table 4. In the matched Ureteroscopy cohorts, operative time was significantly shorter with HU30M compared to 7.5 Fr: 36 (IQR 29–50) vs. 73 (IQR 60–149) minutes, *p* = 0.003. Postoperative hemoglobin, leukocyte and CRP values, as well as renal function parameters, showed no significant differences (Table 5). In the matched ECIRS cohorts, operative times did not differ significantly between HU30M and 7.5 Fr: 57 (IQR 43–63) vs. 50 (IQR 45–57) minutes, *p* = 0.7. Postoperative hemoglobin was lower in the 7.5 Fr group (12.6 vs. 14.1 g/dL, *p* = 0.042), but no significant difference in hemoglobin decrease was observed (*p* = 0.8). A significant difference was observed in postoperative leucocyte levels and leukocyte dynamics, while CRP, creatinine and GFR levels remained comparable (Table 6).

## 4. Discussion

Atraumatic intubation of the ureteral orifice remains a challenge for primary ureterorenoscopy. In many cases, prestenting or dilation of the distal ureter is still required, both of which are associated with significant morbidity. In our initial case series, we evaluated the success rate and morbidity of primary ureterorenoscopy using the world’s thinnest flexible ureterorenoscope—the 6.3 Fr HU30M (HugeMed)—across several clinically relevant scenarios. Atraumatic intubation was achieved in 36 of 38 renal units (95%). During stone treatment, the stone-free rate was 17 of 19 cases (89%). Complication rates were low, with only 2 of 34 patients (6%) experiencing Clavien–Dindo grade II events at most. Postoperative changes in blood loss, inflammatory markers, and renal function were minimal, and no patient required procedure-related readmission within 30 days. Our preliminary series demonstrates that primary ureterorenoscopy with the 6.3 Fr HU30M can be performed safely and atraumatically.

Studies report that primary access of the upper urinary tract by ureterorenoscopy fails in up to one-third of cases due to a narrow ureteral orifice or distal part of the ureter [1]. Instrumental dilation using balloon dilators or sequential dilators carries the risk of ureteral perforation of up to 8% [8]. In a retrospective multicenter study, Hudson et al. evaluated primary access to the upper urinary tract using flexible ureterorenoscopes with shaft diameters of 9 Fr, 8.6 Fr, 8.4 Fr, and 7.4 Fr. They found an inverse relationship between scope size and access success, with smaller-diameter instruments achieving higher primary access rates [9]. Miniaturization has therefore been a primary objective in the technical evolution of digital flexible ureterorenoscopy. In our pilot study, using the world’s thinnest flexible ureterorenoscope—the 6.3 Fr HU30M—we achieved atraumatic access to the upper urinary tract without any need for dilatation or prestenting in 95% of cases.

In contrast, placement of a ureteral stent for approximately one week provides sufficient passive dilation of the ureter, facilitating access to the upper urinary tract in most patients. Prestenting has been shown to increase stone-free rates and render active ureteral dilation unnecessary [10,11]. However, prestenting followed by definitive ureterorenoscopy requires two separate invasive procedures—and if a protective stent is placed postoperatively, a third intervention is added. This “triple hit” not only increases patient discomfort but also raises the risk of stent-related morbidity. Jessen et al. have shown that initial prestenting does not obviate the need for postoperative stenting [10]. By contrast, in our pilot study, we achieved atraumatic primary ureterorenoscopy, allowing us to eliminate prestenting in 95% of cases, thereby reducing the overall procedural burden. Several studies showed a negative impact of ureteral stents on health-related quality of life: up to 80% of stented patients suffer lower urinary tract symptoms, flank pain, or urinary tract infections, and nearly half will miss work because of stent discomfort [12,13]. Moreover, real-world data show that the waiting times for definitive stone treatment after prestenting exceed the necessary seven-day period by months, further compounding costs and patient morbidity [6].

Assessment of the stone-free rate following ureterorenoscopy is challenging due to variations in imaging modality, timing of evaluation, and definitions of clinically significant residual fragments. Nevertheless, depending on size and location of the stone, ureterorenoscopy consistently achieves an excellent stone-free rate [14]. Likewise, in our pilot study, the stone-free rate after primary ureterorenoscopy was comparable at 89%, as confirmed by intraoperative endoscopic and fluoroscopic assessment and by postoperative ultrasound examination.

Intraoperative and early postoperative complications of ureterorenoscopy are classified as major or minor. Major intraoperative events—such as ureteral avulsion, significant ureteral wall injury, or severe hemorrhage—are rare and did not occur in our series. Early postoperative major complications include renal pseudoaneurysm or arteriovenous fistula, urinoma or perirenal hematoma, urosepsis, fever, and urinary tract infection. Transient rises in serum creatinine occur in approximately 1.6% of cases and are usually self-limiting [1]. In our cohort, overall complication rates were low. The median hemoglobin level decreased by 0.8 g/dL, and white blood cell counts rose by a median of 2.5 G/L, while renal function parameters remained unchanged from baseline. Two patients developed postoperative fever after ECIRS with primary ureterorenoscopy, requiring antibiotic therapy (Clavien–Dindo II). However, because nine ECIRS cases combined percutaneous nephrolitholapaxy with primary ureterorenoscopy, the laboratory results cannot be attributed solely to the ureterorenoscopy. Importantly, atraumatic primary ureterorenoscopy using the 6.3 Fr HU30M demonstrated a minimal overall complication rate in our series. Ureteral stricture—a severe long-term complication—is thought to be prevented by ongoing miniaturization of ureterorenoscopic instruments [1]. In our cohort, no patient required re-admission within 30 days of primary URS with the HU30M.

The expanded analyses, including a contemporary control cohort with the standard of care at our department, a 7.5 Fr digital single-use ureteroscope, provided further insights into the relative performance of HU30M. In the ureterorenoscopy setting, HU30M was associated with a significantly shorter operative time compared to 7.5 Fr, consistent with recent findings reporting similar performance [15]. In the ECIRS cohorts, postoperative hemoglobin values were significantly lower in the 7.5 Fr group. However, the difference between pre- and postoperative hemoglobin was not significant. By contrast, postoperative leukocyte counts and leukocyte dynamics were significantly higher in the HU30M group. The clinical relevance of this finding needs to be interpreted with caution. As stated above, the ECIRS HU30M cohort included two cases of postoperative fever. Thus, while a statistical difference in leukocyte dynamics was observed, these cases strongly suggest that infection-related risk factors, rather than the choice of instrument, contributed to the transient febrile episodes. No major complications (Clavien–Dindo ≥ IIIb) occurred in either group.

Several limitations of our study warrant acknowledgement. First, our cohort was heterogeneous, encompassing patients who underwent different therapeutic modalities; this diversity precludes establishing a causal relationship between outcomes and the use of the HU30M ureterorenoscope. Nevertheless, all subgroups shared the same primary endpoint: successful access to the upper urinary tract. Second, stone-free status was determined intraoperatively by endoscopic and fluoroscopic assessment and by postoperative ultrasound within 3 days after surgery. While this approach reflects common clinical practice, it does not represent the most sensitive modality for detecting residual fragments. Among multiple available methods for evaluating stone clearance, the non-contrast CT (NCCT) is widely recognized as the most sensitive technique, but its use is limited by higher cost and radiation exposure. By contrast, ultrasonography avoids radiation but is operator-dependent and less reliable in detecting stones <5 mm, which may result in overestimation of the stone-free rate. Thus, the stone-free rate reported in our study may be slightly higher than would be observed with NCCT-based follow-up. Importantly, however, small residual fragments of 1–2 mm have been suggested to be of limited clinical significance, and therefore the clinical impact of these potential discrepancies remains uncertain [14]. Third, our assessment of the 30-day procedure-related readmission rate provides only a limited evaluation of the risk of ureteral stricture. Fourth, as a single-center study with a limited sample size, our findings may not be generalizable. In the comparative setting, the cohort remains limited. Propensity score matching was performed based on a single variable (maximal stone diameter), limiting robustness. The wide interquartile ranges observed for several outcomes also underline the need for cautious interpretation. Furthermore, in the ureterorenoscopy 7.5 Fr cohort, additional factors such as the use of ureteral access sheaths in 7/14 patients (50%) may have influenced outcomes. Therefore, while our findings suggest procedural efficiency advantages for HU30M and overall comparable safety to the current digital single-use ureteroscope standard, they should be regarded as exploratory and hypothesis-generating. To our knowledge, this is the first case series demonstrating atraumatic primary ureterorenoscopy with the world’s thinnest flexible scope. Larger, multicenter studies will be required to validate and extend these observations.

## 5. Conclusions

The present study demonstrates that primary ureteroscopy with the HU30M is a safe and effective approach for a variety of clinical indications. The high success rates for primary ostial intubation demonstrate the feasibility without prestenting, reducing delays and minimizing the number of necessary procedures for patients. Low complication and readmission rates underscore the safety profile of this approach.

## Figures and Tables

**Table 1:** Clinicopathological characteristics and perioperative outcomes of patients undergoing primary flexible ureteroscopy using the single-use flexible ureteroscope HU30M. Continuous variables are presented as median with interquartile range (IQR), and categorical variables are presented as proportions. Abbreviations: ureteroscopy: flexible ureteroscopy. ECIRS: endoscopic combined intrarenal surgery. BMI: body mass index. ASA: American Society of Anaesthesiologists. HU: Hounsfield units.

Characteristic	All	Diagnostic Ureteroscopy	Stone Treatment (Ureteroscopy Only)	Stone Treatment (ECIRS)
	*n* = 34 (100%)	*n* = 15 (44%)	*n* = 10 (29%)	*n* = 9 (27%)
Age (years)	62 (46–71)	67 (61–80)	50 (41–68)	58 (46–62)
Gender				
Male	17 (50%)	6 (40%)	7 (70%)	4 (44%)
Female	17 (50%)	9 (60%)	3 (30%)	5 (56%)
BMI (kg/m^2^)	27 (22–30)	23 (22–29)	27 (25–32)	27 (23–28)
ASA class >2	14 (41%)	8 (53%)	3 (30%)	3 (33%)
Number of stones	2 (1–3)	-	2 (1–2)	2 (1–4)
Size of biggest stone (mm)				
Coronal plane	8 (6–14)	-	6 (4–6)	15 (13–16)
Sagittal plane	9 (5–14)	-	5 (4–5)	14 (11–18)
Transversal plane	8 (4–13)	-	4 (4–5)	14 (11–15)
Guy’s stone score	-	-	-	3 (2–3)
Radiodensity biggest stone (HU)	800 (492–940)	-	718 (337–897)	830 (714–967)
Stone-free rate	17/19 (90%)	-	9/10 (90%)	8/9 (89%)
Operation time (min)	43 (29–57)	43 (27–61)	31 (29–43)	57 (42–63)
Postoperative stenting	33 (97%)	15 (100%)	10 (100%)	8 (89%)
Duration of postoperative stenting	7 (2–14)	7 (5–14)	7 (7–14)	2 (2–3)
Clavien–Dindo complication grade				
No complications	32 (94%)	15 (100%)	10 (100%)	7 (78%)
Minor complication (≤IIIa)	2 (5.9%)	0 (0%)	0 (0%)	2 (22%)
Major complication (≥IIIb)	0 (0%)	0 (0%)	0 (0%)	0 (0%)
Preoperative urine culture positive	4 (12%)	1 (6.7%)	1 (10%)	2 (22%)
30-day emergency readmission rate	0 (0%)	0 (0%)	0 (0%)	0 (0%)

**Table 2:** Approach of ostial intubation. Of the 34 patients included, four underwent bilateral primary ureteroscopy, giving a total of 38 renal units in which primary ureteroscopy was either performed or attempted.

Ostial Intubation	*n* = 38 Renal Units (100%)
Failure	*n* = 2 (5.3%)
No guidewire (no-touch technique)	*n* = 11 (29%)
One guidewire (parallel to URS)	*n* = 5 (13%)
One guidewire (through URS)	*n* = 9 (24%)
Two guidewires (railway technique)	*n* = 11 (29%)

**Table 3:** Postoperative laboratory parameters and the difference from preoperative baseline values, presented as median with interquartile range (IQR).

	All
Hemoglobin (g/dl)	13.9 (12.5–14.5)
	−0.8 (−1.3, −0.1)
Leukocytes (G/L)	9.2 (7.3–12.3)
	+2.5 (0.4–4.7)
CRP (mg/dl)	0.5 (0.4–1.2)
	+0.4 (0.1–0.6)
Creatinine (mg/dl)	1.0 (0.9–1.1)
	±0 (−0.1–0.1)
GFR (mL/min)	80 (59–92)
	±0 (−12–0)

**Table 4:** Clinicopathological characteristics and perioperative outcomes of patients undergoing primary flexible ureteroscopy and endoscopic combined intrarenal surgery using the single-use flexible ureteroscope HU30M (6,3 Fr) and the standard of care 7.5 Fr single-use flexible ureteroscope.

Characteristic	Overall N = 42 ^1^	Ureteroscopy HU30M N = 10 ^1^	Ureteroscopy 7.5 Fr N = 14 ^1^	ECIRS HU30M N = 9 ^1^	ECIRS 7.5 Fr N = 9 ^1^
Age	60 (46, 69)	50 (39, 69)	64 (58, 73)	58 (46, 62)	60 (55, 65)
Gender					
male	26/42 (62%)	7/10 (70%)	9/14 (64%)	4/9 (44%)	6/9 (67%)
female	16/42 (38%)	3/10 (30%)	5/14 (36%)	5/9 (56%)	3/9 (33%)
BMI	26.9 (21.0, 32.0)	27.0 (24.0, 33.0)	26.7 (19.0, 32.0)	27.0 (23.0, 28.0)	25.2 (20.3, 30.1)
ASA class	2.00 (2.00, 3.00)	2.00 (2.00, 3.00)	2.00 (2.00, 3.00)	2.00 (2.00, 3.00)	2.00 (2.00, 3.00)
Number of stones	2.00 (1.00, 3.00)	1.50 (1.00, 2.00)	2.00 (1.00, 5.00)	2.00 (1.00, 4.00)	2.00 (1.00, 3.00)
Biggest stone					
Coronal Plane	9 (6, 14)	6 (4, 6)	9 (6, 12)	15 (13, 16)	10 (6, 14)
Sagittal Plane	8.5 (5.0, 12.5)	5.0 (4.0, 5.0)	7.0 (6.1, 11.5)	14.0 (11.0, 18.0)	9.0 (6.0, 12.0)
Transversal Plane	8.0 (5.0, 11.5)	4.0 (4.0, 5.0)	9.9 (6.3, 11.5)	14.0 (11.0, 15.0)	7.0 (5.0, 9.0)
Guy’s stone score	2.00 (1.00, 2.00)	1.00 (1.00, 2.00)	-	3.00 (2.00, 3.00)	2.00 (1.00, 2.00)
Radiodensity biggest stone (HU)	830 (599, 1093)	718 (317, 982)	869 (400, 1379)	830 (627, 1036)	1050 (800, 1100)
Stone-free rate	39/42 (93%)	9/10 (90%)	13/14 (93%)	8/9 (89%)	9/9 (100%)
Operation time (min)	52 (41, 75)	36 (29, 50)	66 (48, 99)	57 (43, 63)	50 (45, 57)
Postoperative stenting	38/42 (90%)	10/10 (100%)	14/14 (100%)	8/9 (89%)	6/9 (67%)
Duration postoperative stenting (d)	3 (2, 8)	7 (7, 14)	2 (2, 6)	2 (2, 3)	6 (2, 24)
Clavien–Dindo complication grade					
No complications		10 (100%)	12 (86%)	7 (78%)	9 (100%)
Minor complication (≤IIIa)		0 (0%)	2 (14%)	2 (22%)	0 (0%)
Major complication (≥IIIb)		0 (0%)	0 (0%)	0 (0%)	0 (0%)
Preoperative urine culture positive	14/42 (33%)	1/10 (10%)	7/14 (50%)	3/9 (33%)	3/9 (33%)
30-day emergency readmission rate	0 (0%)	0 (0%)	0 (0%)	0 (0%)	0 (0%)

^1^ Median (Q1, Q3).

**Table 5:** Outcomes of propensity score-matched comparison by maximal stone size between HU30M and 7.5 Fr in the ureteroscopy cohorts. Outcomes include operation duration, postoperative laboratory parameters and the difference from preoperative baseline values.

Outcomes	Ureteroscopy HU30M N = 10 ^1^	Ureteroscopy 7.5 Fr N = 10 ^1^	*p*-Value ^2^
Operation time (min)	36 (29, 50)	73 (60, 149)	**0.003**
Postoperative Hb (g/dL)	13.90 (13.70, 14.40)	13.20 (10.70, 14.20)	0.3
Postoperative Hb difference	−0.65 (−1.10, −0.30)	−1.05 (−1.50, −0.20)	0.9
Postoperative leukocytes (G/L)	8.7 (7.1, 10.1)	11.0 (7.1, 12.2)	0.4
Postoperative leukocyte difference	2.46 (0.02, 3.59)	1.61 (−0.53, 3.83)	0.6
Postoperative CRP (mg/dL)	0.50 (0.50, 0.80)	1.00 (0.30, 1.60)	>0.9
Postoperative CRP difference	0.15 (−0.03, 1.43)	0.10 (0.10, 1.20)	0.9
Postoperative creatinine (mg/dL)	1.00 (0.90, 1.10)	1.05 (1.00, 1.20)	0.4
Postoperative creatinine difference	0.00 (−0.10, 0.10)	0.05 (0.00, 0.10)	0.5
Postoperative GFR (mL/min)	80 (58, 85)	76 (53, 84)	0.7
Postoperative GFR difference	−5 (−13, 0)	−3 (−10, 0)	0.8

^1^ Median (Q1, Q3), ^2^ Wilcoxon rank sum test; Wilcoxon rank sum exact test.

**Table 6:** Outcomes of propensity score-matched comparison by maximal stone size between HU30M and 7.5 Fr in the ECIRS cohorts. Outcomes include operation duration, postoperative laboratory parameters and the difference from preoperative baseline values.

Outcomes	ECIRS HU30M N = 9 ^1^	ECIRS PUSEN N = 9 ^1^	*p*-Value ^2^
Operation time (min)	57 (43, 63)	50 (45, 57)	0.7
Postoperative Hb (g/dL)	14.10 (12.30, 14.90)	12.60 (10.40, 13.00)	**0.042**
Postoperative Hb difference	−0.90 (−1.80, 0.10)	−1.20 (−1.70, −0.70)	0.8
Postoperative leukocytes (G/L)	13.20 (10.10, 13.50)	6.86 (6.64, 10.10)	**0.030**
Postoperative leukocyte difference	5.31 (2.16, 5.93)	0.80 (−0.03, 2.10)	**0.014**
Postoperative CRP (mg/dL)	1.40 (1.10, 2.30)	3.70 (1.00, 7.20)	0.8
Postoperative CRP difference	0.73 (0.53, 1.43)	2.60 (−0.10, 7.60)	0.4
Postoperative creatinine (mg/dL)	1.00 (0.90, 1.10)	1.00 (1.00, 1.10)	>0.9
Postoperative creatinine difference	0.10 (0.00, 0.20)	0.10 (−0.10, 0.20)	0.4
Postoperative GFR (mL/min)	71 (65, 84)	74 (63, 87)	0.7
Postoperative GFR difference	−12 (−23, 0)	−4 (−10, 5)	0.2

^1^ Median (Q1, Q3), ^2^ Wilcoxon rank sum test; Wilcoxon rank sum exact test.

## Data Availability

The data presented in this study are available on request from the corresponding author due to privacy concerns.
